# Chewing lice of Bearded Reedling (*Panurus biarmicus*) and diversity of louse-host associations of birds in reed beds in Slovakia

**DOI:** 10.1051/parasite/2024006

**Published:** 2024-02-08

**Authors:** Oldřich Sychra, Lucie Sušilová, Tomáš Najer, Ivan Literák, Ivo Papoušek, Jana Martinů, Alfréd Trnka, Miroslav Capek

**Affiliations:** 1 Department of Biology and Wildlife Diseases, Faculty of Veterinary Hygiene and Ecology, University of Veterinary Sciences Brno Palackeho tr. 1946/1 61242 Brno Czechia; 2 Department of Veterinary Sciences, Faculty of Agrobiology, Food and Natural Resources, Czech University of Life Sciences Prague Kamycka 129 165 00 Prague 6 Czechia; 3 Illinois Natural History Survey, Prairie Research Institute, University of Illinois Urbana-Champaign IL 61820 USA; 4 Department of Parasitology, Faculty of Science, University of South Bohemia České Budějovice Czechia; 5 Institute of Parasitology, Biology Centre, Czech Academy of Sciences, v. v. i České Budějovice Czechia; 6 Department of Biology, University of Trnava Priemyselna 4 918 43 Trnava Slovakia; 7 Institute of Vertebrate Biology, Czech Academy of Sciences, v. v. i Kvetna 8 603 65 Brno Czechia

**Keywords:** *Menacanthus*, *Penenirmus*, Redescription, Prevalence, Intensity

## Abstract

A total of 1,621 wild birds representing 34 species were examined for chewing lice in reed beds in southwestern Slovakia during the pre-breeding migration 2008–2009 and 2016–2019. A total of 377 (23.3%) birds representing 15 species were parasitized by 26 species of chewing lice of 12 genera*.* Dominant genera were *Penenirmus* (with dominance 32.6%) and *Menacanthus* (29.4%), followed by *Brueelia* (12.6%), *Acronirmus* (10.8%), *Philopterus* (7.7%), and *Myrsidea* (4.2%). We evaluated 33 host-louse associations including both 1) host-generalist, parasitizing more than one host species and host-specific lice, occurring only on a single host species, and 2) lice species with large range geographic distribution, reported across the range of the distribution of their hosts and lice species with only occasional records from a limited area within the range of their hosts. The Bearded Reedling, *Panurus biarmicus* (Linnaeus, 1758), was parasitized by two species of chewing lice, *Menacanthus brelihi* Balát, 1981 and *Penenirmus visendus* (Złotorzycka, 1964), with conspicuously different prevalences (5.6% vs. 58.2%, respectively; *n* = 251). New material enabled us to redescribe both species of lice: the first one is resurrected from previous synonymy as a valid species. A fragment of the mitochondrial cytochrome oxidase I gene was sequenced from these two species in order to assess their relative phylogenetic position within their genera. Our study demonstrates the importance of an adequate identification of parasites, especially on rarely examined and endangered hosts.

## Abstract (fr)

Au total, 1 621 oiseaux sauvages représentant 34 espèces ont été examinés à la recherche de mallophages dans les roselières du sud-ouest de la Slovaquie au cours de la migration de pré-reproduction 2008–2009 et 2016–2019. Parmi ceux-ci, 377 oiseaux (23,3 %), représentant 15 espèces, étaient parasités par 26 espèces de mallophages de 12 genres. Les genres dominants étaient *Penenirmus* (avec une dominance de 32,6 %) et *Menacanthus* (29,4 %), suivis de *Brueelia* (12,6 %), *Acronirmus* (10,8 %), *Philopterus* (7,7 %) et *Myrsidea* (4,2 %). Nous avons évalué 33 associations mallophage-hôte comprenant à la fois 1) des espèces de mallophages généralistes, parasitant plus d’une espèce hôte, et des mallophages spécifiques, présents uniquement sur une seule espèce hôte et 2) des espèces de mallophages ayant une large répartition géographique, signalées à travers l’étendue de la répartition de leurs hôtes, et des espèces de mallophages avec seulement des observations occasionnelles dans une zone limitée à l’intérieur de l’aire de répartition de leurs hôtes. La Panure à moustaches, *Panurus biarmicus* (Linnaeus, 1758), était parasitée par deux espèces de mallophages, *Menacanthus brelihi* Balát, 1981 et *Penenirmus visendus* (Złotorzycka, 1964), avec des prévalences nettement différentes (respectivement 5,6 % et 58,2 %, *n* = 251). Du nouveau matériel nous a permis de redécrire les deux espèces de mallophages, la première étant ressuscitée de la synonymie précédente en tant qu’espèce valide. Un fragment du gène mitochondrial de la cytochrome oxydase I a été séquencé à partir de ces deux espèces afin d’évaluer leur position phylogénétique relative au sein de leurs genres. Notre étude démontre l’importance d’une identification adéquate des parasites, en particulier sur les hôtes rarement examinés et menacés.

## Introduction

A diverse assemblage of chewing lice (Phthiraptera) and their host associations are well-known from Slovakia. Recently we reviewed all available published records, old museum collections, and recently collected material of chewing lice from Slovakia and provided a checklist containing 249 species of chewing lice – 65 amblyceran species from 22 genera representing the families Laemobothriidae, Menoponidae, and Ricinidae, and 184 ischnoceran species from 54 genera of the family Philopteridae – and 358 host-louse associations from 171 bird species from 21 orders [36]. Nevertheless, information about infestation characteristics like prevalence or mean intensity is still scarce, incomplete, and focused on louse species from hosts occurring at higher elevations, in montane forests and shrublands [2, 8, 17–20].

We recently focused on a lowland wetland bird community. We evaluated infestation characteristics of ectoparasites in relation to the migration behavior and sexual dimorphism of five passerine bird species that represent habitat specialists breeding in this environment dominated by Common Reed (*Phragmites australis*) [51]. In addition to habitat specialists, i.e., *Acrocephalus* spp., *Locustella luscinioides* (Savi, 1824), and *Panurus biarmicus* (Linnaeus, 1758), the avifauna of the reed beds also includes birds that use this environment to roost during both the breeding season and migration, e.g., *Hirundo rustica* Linnaeus, 1758, *Motacilla alba* Linnaeus, 1758, and *Sturnus vulgaris* Linnaeus, 1758 [53].

The Bearded Reedling, *Panurus biarmicus,* has a wide range across the Palearctic realm. It is evaluated as Least Concern in the Red List Assessment [7]. The distribution of the species has seen significant gains elsewhere across its European range [26], but the populations in Slovakia have decreased within the last few years [6] and they are considered as Near Threatened there [10].

We focused on the lice of *P. biarmicus*, and only two species of chewing lice are known from this host – *Menacanthus eurysternus* (Burmeister, 1848) and *Penenirmus visendus* (Złotorzycka, 1964) [40]. *Menacanthus* lice were first reported from *P. biarmicus* by Balát [4] who described them as *Menacanthus brelihi* Balát, 1981. Balát [4] included one picture of male parameres and photographs of the holotype female and paratype male, with a short general text description. Consequently, Krištofík [29] examined the Balát’s type material and stated that “examined slides are of bad quality”. He concluded his re-examination of this material with the statement that all the main features of examined specimens were identical to *Me. eurysternus*. As a result, he synonymized *Me. brelihi* with *Me. eurysternus. Penenirmus visendus* was originally described as *Panurinirmus visendus* by Złotorzycka [55] based on a single female from the Bearded Reedling from Poland. Later, Emerson [11] synonymized *Panurinirmus* with *Penenirmus*. To date, no description of the male of this species has been published.

In this paper, we extend the knowledge of ectoparasites of passerine birds occurring in reed bed ecosystems. The aims of this paper are to: (1) present new data on the species distribution of chewing lice found on birds in reed beds in southwestern Slovakia; (2) clarify information on their infestation characteristics; (3) redescribe both sexes of *Penenirmus visendus* and *Menacanthus brelihi* and resurrect the latter as a valid taxon; and (4) confirm the validity of these taxa also by phylogenetic analysis of a fragment of the mitochondrial cytochrome oxidase I (*COI*) gene.

## Materials and methods

Birds were captured by mist-netting in reed beds of the National Nature Reserve Parížske močiare located near the villages of Gbelce and Nová Vieska (47°52′ N, 18°30′ E) in southwestern Slovakia. For more details about habitat see Kloubec *&* Capek [28], about period see Sychra *et al.* [51]. The fumigation chamber method was applied to collect lice from the birds, using chloroform as a fumigant for 7–10 min, complemented by visual examination of the head. Louse identification was based on papers by Gustafsson & Bush [14], Gustafsson *et al.* [15], Najer *et al.* [35], Palma & Price [37], Price [39], Rheinwald [43], Sychra *et al.* [47, 49–50], and Złotorzycka [56–58]. The taxonomy of birds follows Gill *et al.* [13].

Measurements were made in QuickPHOTO MICRO 3.1 (Promicra, Prague, Czechia). In the following redescriptions, all measurements are given in millimeters for the following dimensions: ANW = female anus width; AW = abdomen width [at level of segment IV (for *Menacanthus*) or V (for *Penenirmus*)]; GSL = male genital sac sclerite length; GW = male genitalia width; HL = head length (at midline); HW = head width (at temples); MW = metathorax width; POW = preocular width; PTW = pterothorax width; PW = prothorax width; TL = total length.

The specimens examined are deposited at the Moravian Museum, Brno, Czechia (MMBC), Museum of Natural History, University of Wrocław, Poland (MNHW), and at the Department of Biology and Wildlife Diseases, University of Veterinary Sciences Brno, Czechia (UVSB).

Infestation characteristics were counted as in Sychra *et al.* [46, 48]. We used the following categories to designate the infestation on passerine hosts: very light infestation (1–10 lice per bird); light infestation (11–20 lice); medium infestation (21–30 lice); heavy infestation (31–50 lice); very heavy infestation (51–100 lice).

Sequences of a 379 bp fragment of the *COI* gene were obtained from *Menacanthus brelihi* and from *Penenirmus visendus* from *Panurus biarmicus* using methods described by Johnson *et al.* [22]. New sequences (GenBank accession numbers OR533291–OR533294, OR626644–OR626646) were aligned together with all available sequences from *Menacanthus* and *Penenirmus* genera previously published in the literature and deposited in GenBank [21–24, 31–32, 34, 49, 52] using Geneious 9.1.8 [25] in order to assess their genetic divergence and interspecific relationships. For phylogenetic analysis, we first computed the Akaike information criterion (AIC) computed in MEGA 7.0.14 [30] to identify the most appropriate model of nucleotide substitution. The phylogenetic tree was built with the Bayesian inference analysis (BI) using the Mr. Bayes 3.2.6 plugin in Geneious 9.1.8 [25, 44] with a GTR + G + I model for 10(7) generations, with trees sampled every 1,000 generations. A majority rule consensus tree was summarized after discarding 1,000 trees as a burn-in. Computation of genetic *p*-distances was performed in MEGA 7.0.14 [30].

## Results

A total of 1,621 wild birds representing 34 species were examined for chewing lice (Table 1). A total of 377 (23.3%) birds representing 15 species were parasitized by 26 species of chewing lice (Table 1)*.* A total of 33 louse-host associations were found, which represented more than 1/2 of the known louse-host associations (*n* = 56) for these 26 bird species examined within their range of distribution [36, 40]. Most birds, *i.e.*, 341 (90.5%, *n* = 377), showed only very light (1–10 lice/host; 76.7%) to light infestations (11–20 lice/host; 13.8%). Medium (21–30 lice/host) and heavy infestation (31–40 lice/host) were recorded on 14 (3.7%) and 16 (4.2%) birds, respectively. The highest infestations were found on two *Hirundo rustica* parasitized by 107 and 66 individuals of *Acronirmus gracilis* (Burmeister, 1838), two *Panurus biarmicus* parasitized by 71 and 61 individuals of *Penenirmus visendus*, one *Acrocephalus schoenobaenus* (Linnaeus, 1758) parasitized by 64 individuals of *Menacanthus curuccae* (Schrank, 1776), and one *Sturnus vulgaris* parasitized by 51 individuals of *Brueelia nebulosa* (Burmeister, 1838). Five of these birds were examined between April 13 and May 1, while one *P. biarmicus* was examined in October.

**Table 1:** List of hosts and their chewing lice. P/E = prevalence = number of birds parasitized (P)/number of birds examined (E); MI = mean intensity = number of individuals of a particular chewing louse species on infested hosts; MA = mean abundance = number of individuals of a particular chewing louse species on examined birds; Σ = a total number of collected lice; N = nymphs; M = Menoponidae; P = Philopteridae; R = Ricinidae.

Bird species	E	P	P/E	MI	MA	Σ	♀	♂	N
Chewing louse family/species			(%)						
**PASSERIFORMES**									
**Family Acrocephalidae**									
*Acrocephalus arundinaceus* (Linnaeus, 1758)	107	0	–	–	–	–	–	–	–
*Acrocephalus paludicola* (Vieillot, 1817)	6	0	–	–	–	–	–	–	–
***Acrocephalus melanopogon* (Temminck, 1823)**									
P/*Philopterus acrocephalus* Carriker, 1949	32	31	96.9	5.3	5.1	164	56	46	62
***Acrocephalus scirpaceus* (Hermann, 1804)**									
M/*Menacanthus curuccae* (Schrank, 1776)	433	46	10.6	5.3	0.6	246	48	10	188
P/*Brueelia* sp.	433	5^1^	1.2	2.0	0.02	10	2	0	8
P/*Philopterus* sp.	433	2	0.5	1.0	0.005	2	1	1	0
***Acrocephalus schoenobaenus* (Linnaeus, 1758)**									
M/*Menacanthus curuccae* (Schrank, 1776)	363	27	7.4	11.4	0.8	308	53	14	241
P/*Brueelia vaneki* Balát, 1981	363	5^2^	1.4	3.2	0.04	16	1	0	15
P/*Philopterus* sp.	363	1	0.3	1.0	0.003	1	0	0	1
**Family Emberizidae**									
***Emberiza schoeniclus* (Linnaeus, 1758)**									
M/*Menacanthus chrysophaeus *(Kellogg, 1896)	32	2	9.4	2.7	0.3	8	1	2	5
R/*Ricinus fringillae* De Geer, 1778	32	1	3.1	3.0	0.1	3	0	0	3
P/*Brueelia blagovescenskyi* Balát, 1955	32	3^3^	9.4	4.3	0.4	13	5	0	8
P/*Philopterus citrinellae* (Schrank, 1776)	32	4	12.5	1.5	0.2	6	3	1	2
**Family Fringillidae**									
***Chloris chloris* (Linnaeus, 1758)**									
P/*Philopterus citrinellae* (Schrank, 1776)	45	3	6.7	2.7	0.2	8	3	1	4
*Linaria cannabina* (Linnaeus, 1758)	5	0	–	–	–	–	–	–	–
*Serinus serinus* (Linnaeus, 1766)	1	0	–	–	–	–	–	–	–
**Family Hirundinidae**									
***Hirundo rustica* Linnaeus, 1758**									
M/*Myrsidea rustica* (Giebel, 1874)	100	23	23.0	1.7	0.4	38	17	10	11
P/*Acronirmus gracilis* (Burmeister, 1838)	100	12^4^	12.0	21.8	2.6	261	88	37	136
P/*Philopterus microsomaticus* Tandan, 1955	100	4^5^	4.0	1.3	0.1	5	1	2	2
***Riparia riparia* (Linnaeus, 1758)**									
M/*Machaerilaemus clayae* (Balát, 1966)	47	2	4.3	2.5	0.1	5	2	0	3
M/*Myrsidea latifrons* (Carriker [& Shull], 1910)	47	2	4.3	1.0	0.04	2	1	0	1
P/*Philopterus microsomaticus* Tandan, 1955	47	1^6^	2.1	1.0	0.02	1	0	1	0
**Family Locustellidae**									
***Locustella luscinioides* (Savi, 1824)**									
M/*Menacanthus obrteli* Balát, 1981	116	20	17.2	4.5	0.8	90	36	13	41
P/*Brueelia locustellae* Fedorenko, 1975	116	5^7^	4.3	14.8	0.6	74	12	5	57
**Family Motacillidae**									
*Motacilla alba* Linnaeus, 1758	2	0	–	–	–	–	–	–	–
*Motacilla flava* Linnaeus, 1758	2	0	–	–	–	–	–	–	–
**Family Muscicapidae**									
*Erithacus rubecula* (Linnaeus, 1758)	1	0	–	–	–	–	–	–	–
*Luscinia svecica* (Linnaeus, 1758)	1	0	–	–	–	–	–	–	–
*Phoenicurus ochruros* (Gmelin, 1774)	1	0	–	–	–	–	–	–	–
*Saxicola rubetra* (Linnaeus, 1758)	2	0	–	–	–	–	–	–	–
*Saxicola rubicola* (Linnaeus, 1766)	1	0	–	–	–	–	–	–	–
**Family Panuridae**									
***Panurus biarmicus* (Linnaeus, 1758)**									
M/*Menacanthus brelihi* Balát, 1981	251	14^8^	5.6	2.1	0.1	29	8	2	19
P/*Penenirmus visendus* (Złotorzycka, 1964)	251	146	58.2	10.0	5.8	1461	237	210	1014
**Family Paridae**									
*Cyanistes caeruleus* (Linnaeus, 1758)	1	0	–	–	–	–	–	–	–
***Parus major* Linnaeus, 1758**									
M/*Menacanthus sinuatus* (Burmeister, 1838)	3	1	1/3	2.0	0.7	2	1	1	0
**Family Passeridae**									
*Passer domesticus* (Linnaeus, 1758)	1	0	–	–	–	–	–	–	–
***Passer montanus* (Linnaeus, 1758)**									
M/*Myrsidea quadrifasciata* (Piaget, 1880)	3	1	1/3	3.0	1.0	3	1	2	0
P/*Rostrinirmus ruficeps* (Nitzsch [in Giebel], 1866)	2	1	1/2	1.0	0.5	1	0	0	1
**Family Sturnidae**									
***Sturnus vulgaris* Linnaeus, 1758**									
M/*Menacanthus eurysternus* (Burmeister, 1838)	44	16^9^	36.4	3.4	1.2	54	21	3	30
M/*Myrsidea cucullaris* (Nitzsch, 1818)	44	9	20.5	6.6	1.3	59	10	22	27
P/*Brueelia nebulosa* (Burmeister, 1838)	44	28	63.6	6.9	4.4	192	74	61	57
P/*Sturnidoecus sturni* (Schrank, 1776)	44	6	13.6	1.8	0.3	11	1	7	3
**Family Sylviidae**									
*Curruca communis* (Latham, 1787)	3	0	–	–	–	–	–	–	–
*Curruca curruca* (Linnaeus, 1758)	3	0	–	–	–	–	–	–	–
*Sylvia atricapilla* (Linnaeus, 1758)	5	0	–	–	–	–	–	–	–
**Family Troglodytidae**									
***Troglodytes troglodytes* (Linnaeus, 1758)**									
P/*Penenirmus albiventris* (Scopoli, 1763)	1	1	1/1	8.0	8.0	8	0	1	7
**Family Turdidae**									
*Turdus philomelos* Brehm, 1831	2	0	–	–	–	–	–	–	–
**CHARADRIIFORMES**									
**Family Scolopacidae**									
*Lymnocryptes minimus* (Brünnich, 1764)	1	0	–	–	–	–	–	–	–
**GRUIFORMES**									
**Family Rallidae**									
***Rallus aquaticus* Linnaeus, 1758**									
P/*Rallicola cuspidatus* (Scopoli, 1763)	1	1	1/1	41.0	41.0	41	13	18	10
P/*Fulicoffula* sp.	1	1	1/1	2.0	2.0	2	0	0	2
**PICIFORMES**									
**Family Picidae**									
*Dendrocopos major* (Linnaeus, 1758)	1	0	–	–	–	–	–	–	–
***Dendrocopos syriacus* (Hemprich & Ehrenberg, 1833)**									
P/*Penenirmus auritus* (Scopoli, 1763)	2	2	2/2	13.5	13.5	27	2	0	25
Total	1621	377	23.3	8.4	1.9	3151	698	470	1983

1 Note: 2 out of 5 hosts with *Brueelia* also harbored *Menacanthus.*

2 Note: 2 out of 5 hosts with *Brueelia* also harbored *Menacanthus.*

3 Note: 1 out of 3 hosts with *Brueelia* harboured also *Menacanthus.*

4 Note: 5 out of 12 hosts with *Acronirmus* also harbored *Myrsidea.*

5 Note: 1 out of 4 hosts with *Philopterus* also harbored *Myrsidea.*

6 Note: a single host with *Philopterus* also harbored *Myrsidea.*

7 Note: 1 out of 5 hosts with *Brueelia* also harbored *Menacanthus.*

8 Note: all 14 hosts with *Menacanthus* also harbored *Penenirmus.*

9 Note: following co-occurrence of collected starling louse species were recorded: *Brueelia*+*Menacanthus* (8 birds), *Brueelia*+*Sturnidoecus* (4), *Brueelia*+*Myrsidea* (3), *Menacanthus*+*Sturnidoecus* (1), *Menacanthus*+*Myrsidea* (1), *Menacanthus+Brueelia+Myrsidea* (1), *Menacanthus+Brueelia +Sturnidoecus* (1).

The majority of birds, *i.e.*, 332 (88%, *n* = 377), were parasitized by only one species of chewing louse; the co-occurrence of two species of lice was recorded from only 43 birds. In 38 cases, co-occurrence of one ischnoceran and one amblyceran louse species was found, in four cases two species of ischnoceran lice were recorded, and in only one case co-inhabitance of two species of amblyceran lice was recorded (Table 1). Co-occurrence of three species was recorded only on two *Sturnus vulgaris*. Almost all species of chewing lice were found only on one host species, with the exception of *Me. curuccae, Philopterus citrinellae* (Schrank, 1776), and *Philopterus microsomaticus* Tandan, 1955, which were recorded on two species of birds (Table 1). The proportion of individuals belonging to twelve genera of lice is ranked as follows: *Penenirmus* (32.6%), *Menacanthus* (29.4%), *Brueelia* (12.6%), *Acronirmus* (10.8%), *Philopterus* (7.7%), *Myrsidea* (4.2%), *Rallicola* (1.7%), *Sturnidoecus* (0.5%), *Machaerilaemus* (0.2%), *Ricinus* (0.1%), *Fulicoffula* (0.1%), and *Rostrinirmus* (0.04%, *n* = 2420). Here we did not include data about 706 *Penenirmus visendus* and 25 *Menacanthus brelihi* from Bearded Reedlings collected in 2019, when collections were focused only on lice from this host.

### Chewing lice of the Bearded Reedling (*Panurus biarmicus*)

#### Redescriptions

Order: Psocodea Hennig, 1966

Suborder: Troctomorpha Roesler, 1944

Infraorder: Phthiraptera Haeckel, 1896

Parvorder: Amblycera Kellogg, 1896

Family: Menoponidae Mjöberg, 1910

Genus: *Menacanthus* Neumann, 1912

### *Menacanthus brelihi* Balát, 1981

Figures 1–2

**Figure 1:**
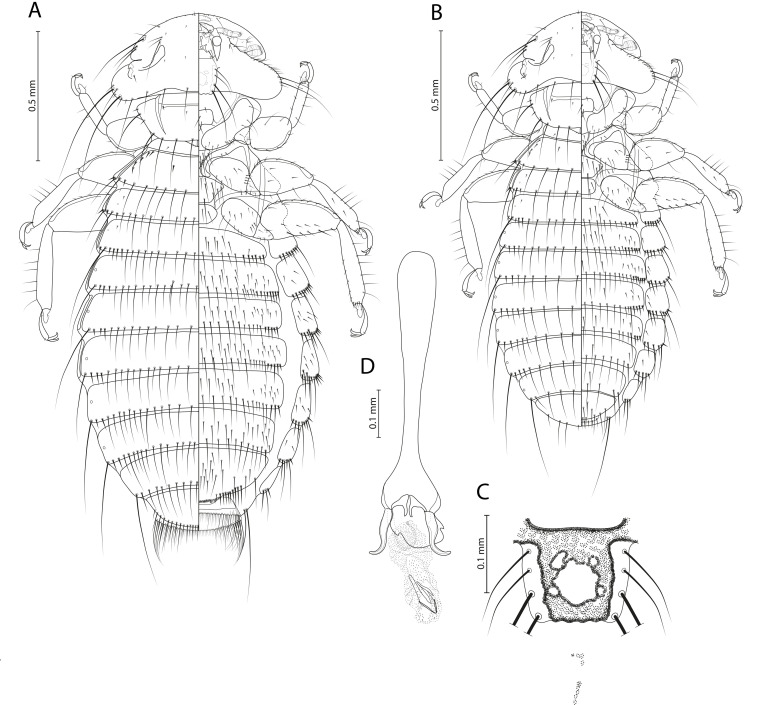
*Menacanthus brelihi* Balát, 1981. (A) Female dorso-ventral view; (B) Male dorso-ventral view; (C) Detail of gula; (D) Male genitalia, dorsal view.

**Figure 2:**
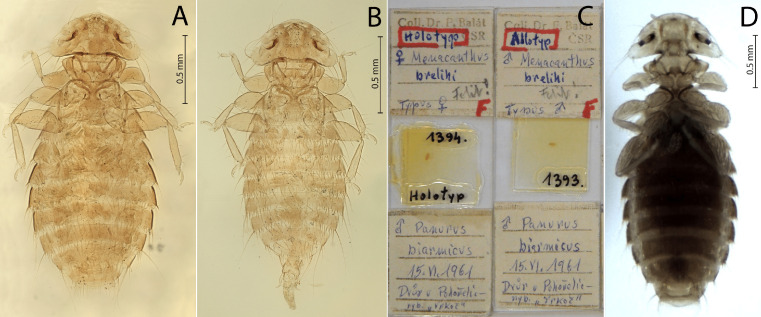
*Menacanthus brelihi* Balát, 1981*.* (A) Holotype female; (B) Allotype male; (C) Type slides; (D) Non-type female.

*Menacanthus brelihi* Balát, 1981: 273: fig. 1, Plate I, Figures 1–2 [4].

**Type host**: *Panurus biarmicus* (Linnaeus, 1758) – Bearded Reedling (Panuridae).

**Type locality**: Velký Dvůr near Pohořelice (south Moravia), Czechia

**Remarks**. *Menacanthus brelihi* belongs to the *curuccae* species group (sensu Martinů *et al.* [32]). Both sexes of this species are readily identified by combination of characters as follows: (1) each side of metanotum with 3–5 lateroanterior setae; (2) characteristic shape of gular plate, with large central lighter “hole” and several small anterior ones, posterior margin straight or undulated, all four setae on each side are inserted in clear lateral area; (3) ocular seta 19 finer than outer central pronotal seta 1; (4) pleurites with anterior setae; (5) large number of sternal setae, especially on sternites III–VI of females (each with more than 53 setae) and sternites III–V of males (each with more than 34 setae); and (6) quite large size. In the key by Price [39], the female of *Me. brelihi* would key out to couplet 28, being closest to *Me. robustus* (Kellogg, 1896). However, the female of *Me. brelihi* can be distinguished from that of *Me. robustus* by a different number of setae on tergites VII and IX (28–29 and 23–26 vs. 23–26 and 32–33) and sternites IV–V (67–72 and 63–75 vs. 60–65 and 51–56). Although males of *Me. robustus* are unknown, the male of *Me. brelihi* would key out to couplet 35 in the key by Price [39], being closest to *Me. tenuifrons* Blagoveshtchensky, 1940. However, the male of *Me. brelihi* can be distinguished from that of *Me. tenuifrons* by a different number of setae on tergite IX (7–8 vs. 10–11) and sternites IV–VII (a total of 114–143 vs. 97–118).

In order to add *Me. brelihi* to Price’s [39] key, the following alterations should be made:

28. Temple width at least 0.57, metathorax width at least 0.56; both anal fringes of over 50 setae; ventral spinous head process at least 0.08 long ......................28a

– Temple width not over 0.56, metathorax width not over 0.55; either or both anal fringes of under 50 setae; ventral spinous head process shorter as long as above ….........29

And insert new couplet among couplets 28 and 29 as follows:

28a. Tergite VII with at least 28 setae, tergite IX with not more than 26 setae; sternite IV with at least 67 setae, sternite V with at least 63 setae …*Me. brelihi*

– Tergite VII with not more than 26 setae, tergite IX with at least 32 setae; sternite IV with not more than 65 setae, sternite V with not more than 56 setae …*Me. robustus*

35. Sternites III–IV each with over 30 setae; subgenital plate with at least 14 setae ................35a

– Sternites III–IV each with under 30 setae; subgenital plate with only up to 12 setae …*Me. sinuatus*

And insert new couplet among couplets 35 and 36 as follows:

35a. Tergite IX with not more than 8 setae and sternites IV–VII with a total of 114–143 setae …*Me. brelihi*

– Tergite IX with at least 10 setae and sternites IV–VII with a total of 97–118 setae …*Me. tenuifrons*

**Figure 3:**
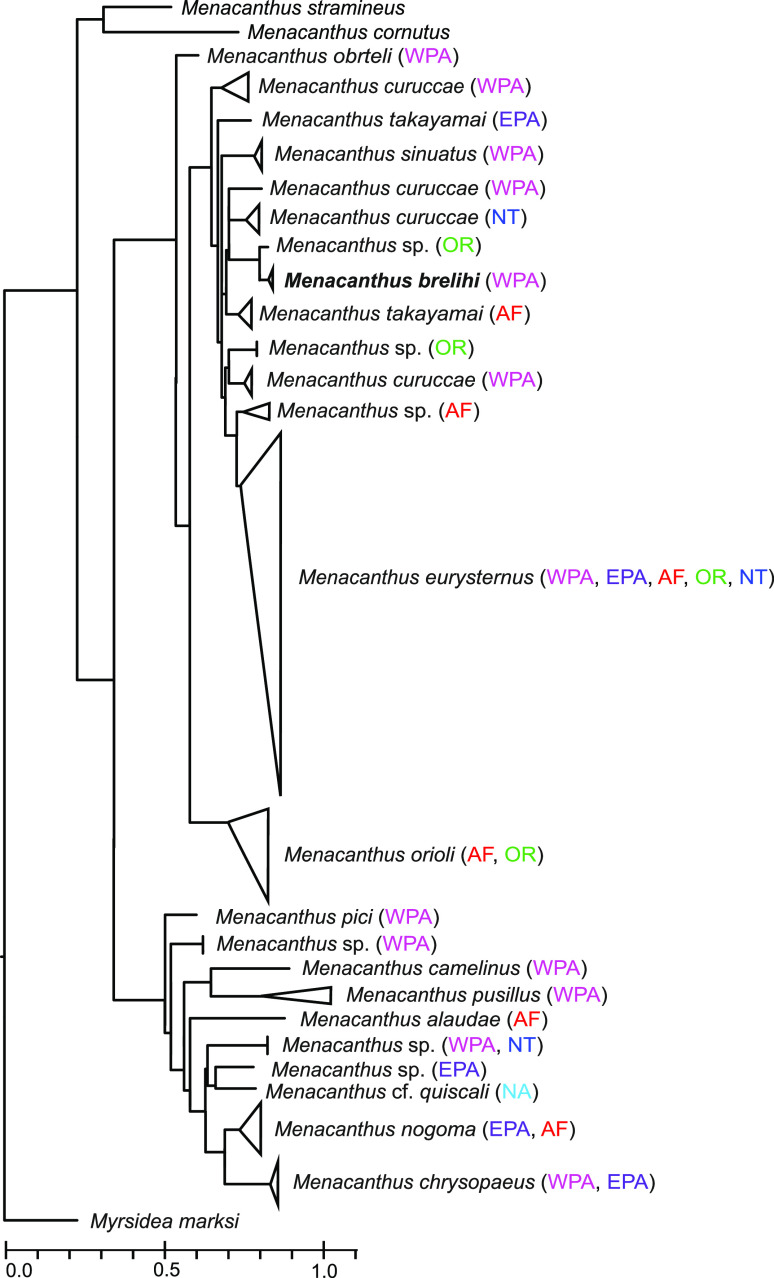
Diagram of the evolution of the *Menacanthus* lineages. The topology was adapted from the cytochrome oxidase subunit I phylogeny in Supplementary Figure S1. The origin of the samples is in parentheses: AF – Afrotropical realm; EPA – Eastern Palearctic realm; NA – Nearctic realm; NT – Neotropical realm; OR – Oriental realm; WPA – Western Palearctic realm.

**Supplementary Figure S1:**
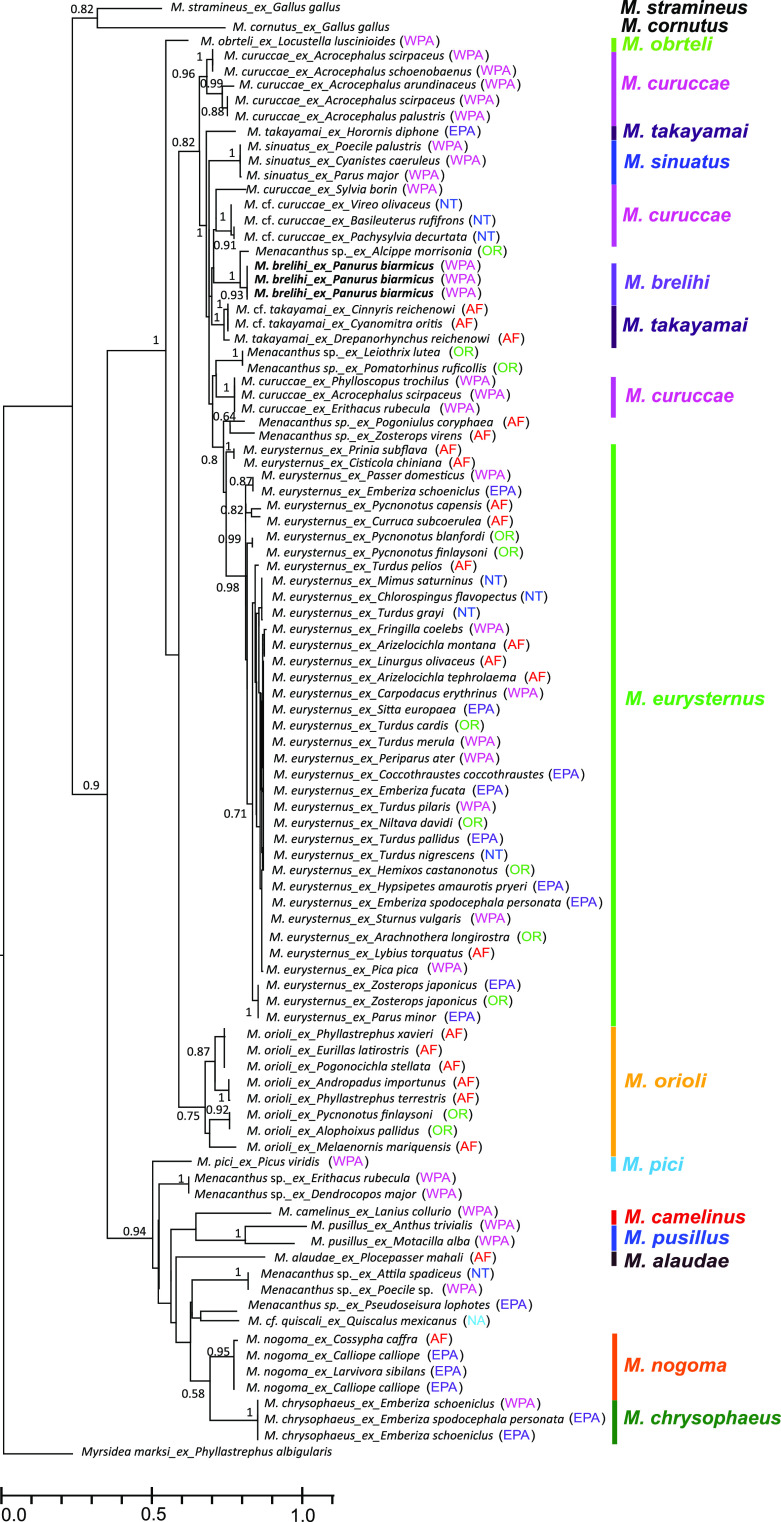
Phylogenetic tree of the *Menacanthus* species based on partial *COI* sequences, estimated with Bayesian analysis based on a 379 bp alignment of a *COI* gene fragment. The tree is drawn to scale, with branch lengths measured in the number of substitutions per site. The numbers above the branches indicate Bayesian posterior probabilities. New sequences are in bold type. — *Colors*: purple – samples from the Western Palearctic realm; dark purple – samples from the Eastern Palearctic realm; red – samples from the Afrotropical realm; green – samples from the Oriental realm; light blue – samples from the Nearctic realm; dark blue – samples from the Neotropical realm.

A fragment of the *COI* gene was sequenced from three specimens of *Me. brelihi* (GenBank accession numbers OR626644–OR626646). Compared to all available sequences of the *Menacanthus* genus, our sequences clustered together with lice of *Me. takayamai* with a sequence divergence of 14–16%. These sequence divergences are large enough to confirm *Me. brelihi* as a separate species. Phylogenetic relationships among sequences obtained from *Me. brelihi* and sequences from other *Menacanthus* species are presented in Figure 3 and Supplementary Figure S1.

**Female** (*n* = 7). As in Figures 1A and 2A. Head with rounded anterior margin, preocular slit, hypopharyngeal sclerites weakly developed and ventral spinous process 0.08–0.09 mm long. Gular plate pigmented, with large central lighter “hole” and several small anterior ones, posterior margin straight or undulated, all four setae on each side are inserted in clear lateral area (Figs. 1C,  2D). Long occipital setae 21, 22, and 23, with alveoli in straight line. Head seta 24 0.20–0.25 mm long. Ocular seta 19 fine 0.02–0.03 mm long. Outer central pronotal setae short and stout. Pronotal margin with 10 long and 4 short setae; prosternal plate moderately developed. Metanotum with 3–5 anterolateral setae on each side and 15–16 marginal setae; mesosternal plate with 10–14 setae; metasternal plate with 12–14 setae. Tergal setae: I, 22–24; II, 24–30, III, 28–31, IV, 26–31, V, 30–34, VI, 28–33; VII, 28–29; VIII, 17–19; IX, 23–26. Pleurites III–VI with 3–5 anterior setae. Sternal setae: I, 3–5; II, 38–46; III, 56–69; IV, 67–72; V, 63–75; VI, 53–63; VII, 41–53; subgenital plate, 25–29, vulval margin with 20–23 setae and prominent deep serrations medioposteriorly. With medioanterior setae on sternites II–VII. Only one very long on each side of posterior margin of abdomen extending beyond ends of anal fringe setae. Ventral and dorsal anal fringes of 57–60 setae.

Dimensions (data for the holotype are in parentheses): POW = 0.47–0.51 (0.50); HW = 0.63–0.65 (0.66); PW = 0.44–0.49 (0.49); MW = 0.59–0.66 (0.64); AW = 0.83–0.93 (0.84); ANW = 0.28–0.31 (0.29); TL = 1.79–2.07 (1.94).

**Male** (*n* = 6). As in Figures 1B and 2B similar to female. Head seta 24 0.17–0.18 mm long. Metanotum with 8–12 marginal setae; mesosternal plate with 8–10 setae; metasternal plate with 10–14 setae. Tergal setae: I, 13–15; II, 17–21; III, 20–22; IV, 19–22; V, 18–21; VI, 17–22; VII, 15–18; VIII, 10–11; IX, 7–8. Pleurites III–VI with 1–2 anterior setae. Sternal setae: I, 2–3; II, 26–38; III, 36–52; IV, 39–47; V, 34–40; VI, 25–33; VII, 16–23; VIII, 8–12; subgenital plate, 6–7 plus 10–11 on posterior margin. Genitalia as on Figure 1D.

Dimensions (data for the allotype are in parentheses): POW = 0.42–0.44 (0.45); HW = 0.54–0.57 (0.56); PW = 0.37–0.39 (0.39); MW = 0.45–0.48 (0.52); AW = 0.59–0.62 (0.66); GW = 0.08–0.10 (0.12); TL = 1.40–1.47 (1.56).

**Examined material. Holotype** ♀ ex *Panurus biarmicus*, Velký Dvůr near Pohořelice (south Moravia), Czechia, 15 Jun. 1961, Balát’s collection no. FB1394 (MMBC, Fig. 2C). **Paratypes:** 1♂ (FB1393, noted as Allotype), 1♀ (FB1395), same data as holotype; 1♀ (FB1225) Nová Ves near Pohořelice (south Moravia), Czechia, 18 Jul. 1962; 3♀♀ from the same host, Neusiedl am See, Austria, 17–18 Sep. 1960 (FB1230, 1234, 1235), leg. Balát (MMBC).

Other material. Non-types ex *P. biarmicus*: 4♀♀, 4♂♂, Gbelce, Slovakia, 16–18 Jul. 2019, leg. Sychra & Ošlejšková (UVSB).

Note: Balát’s collection at MMBC includes seven slides with *Me. brelihi*. According to Balát’s notes, all three specimens from the type series were collected from an adult male of *P. biarmicus*. Although Balát [4] mentioned a total of 11 lice (1♂, 3♀♀ and 7 nymphs) from the adult female of the type host that were examined at Nová Ves, only one slide with one female and one nymph is present at the MMBC. Similarly, according to Balát’s notes, specimens from Austria were collected from two females (2♀♀ from 17 Sep. 1960) and one male of *P. biarmicus* (1♂, 4♀♀ and 2 nymphs from 18 Sep. 1960), but only three females had been mounted on the slide, while other specimens were stored in ethanol. Except for the specimens mentioned within the examined material, all other specimens are missing from the MMBC and must be regarded as lost.

Parvorder: Ischnocera Kellogg, 1896

Family: Philopteridae Burmeister, 1838

Genus: *Penenirmus* Clay & Meinertzhagen, 1938

### *Penenirmus visendus* (Złotorzycka, 1964)

Figures 4–6

**Figure 4:**
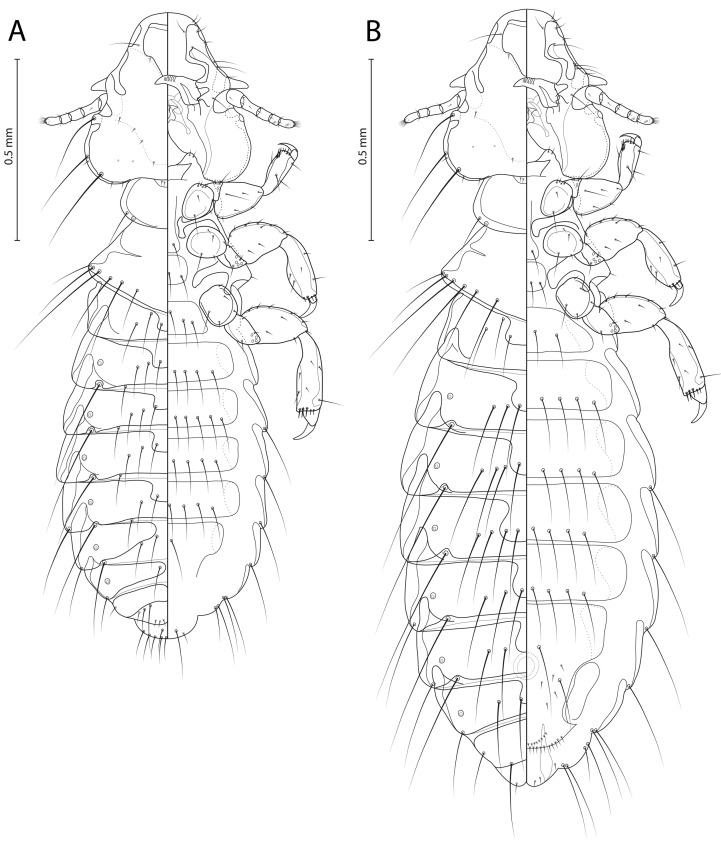
*Penenirmus visendus* (Złotorzycka, 1964)*.* (A) Male dorso-ventral view; (B) Female dorso-ventral view.

**Figure 5:**
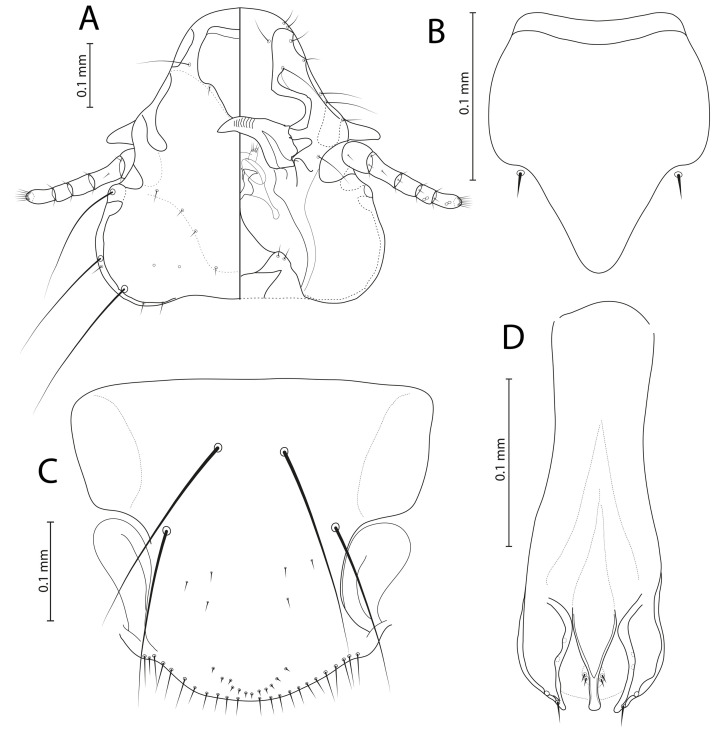
*Penenirmus visendus* (Złotorzycka, 1964)*.* (A) Male head dorso-ventral view; (B) Dorsal anterior head plate; (C) Female subgenital plate and vulval margin, ventral view; (D), Male genitalia dorsal view.

**Figure 6:**
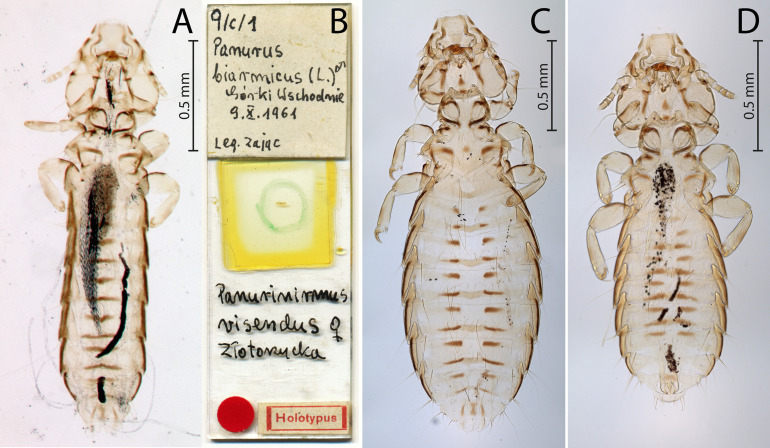
*Penenirmus visendus* (Złotorzycka, 1964)*.* (A) Holotype female; (B) Holotype slide; (C) Non-type Female; (D) Non-type male.

*Panurinirmus visendus* Złotorzycka, 1964: 270: fig. 8d, photo 15 [55].

*Penenirmus visendus* Emerson, 1972: 111 [11].

*Penenirmus visendus* Price, *et al.* [in Price *et al.*], 2003 [40].

**Type host**: *Panurus biarmicus* (Linnaeus, 1758) – Bearded Reedling (Panuridae).

**Type locality**: Górki Wschodnie near Gdańsk, Poland

**Remarks**. Złotorzycka [55] provided a very poor description of *P. visendus* under the name *Panurinirmus visendus* based on a single female from *P. biarmicus* from Poland. There is no comprehensive morphological revision of *Penenirmus* from passerine birds and that is one reason why species determination is difficult and a common practice is to identify species based on host records. To date, 11–14 species of *Penenirmus* have been reported from Europe [33, 40, 57]. To partially address the difficulties in identifying lice in this genus, we compare *P. visendus* with *P. albiventris* a recently well-described by Sychra *et al.* [49]. We found only limited morphological differences between these two species concerning mainly slight differences in the shape of the dorsal anterior head plate (with short blunt posterior process in *P. visendus* compared with quite long and pointed process in *P. albiventris*) and abdominal tergites II–VI (joined by two narrow conspicuously pigmented strips compared with tergites that are joined by a single pigmented strip).

**Figure 7:**
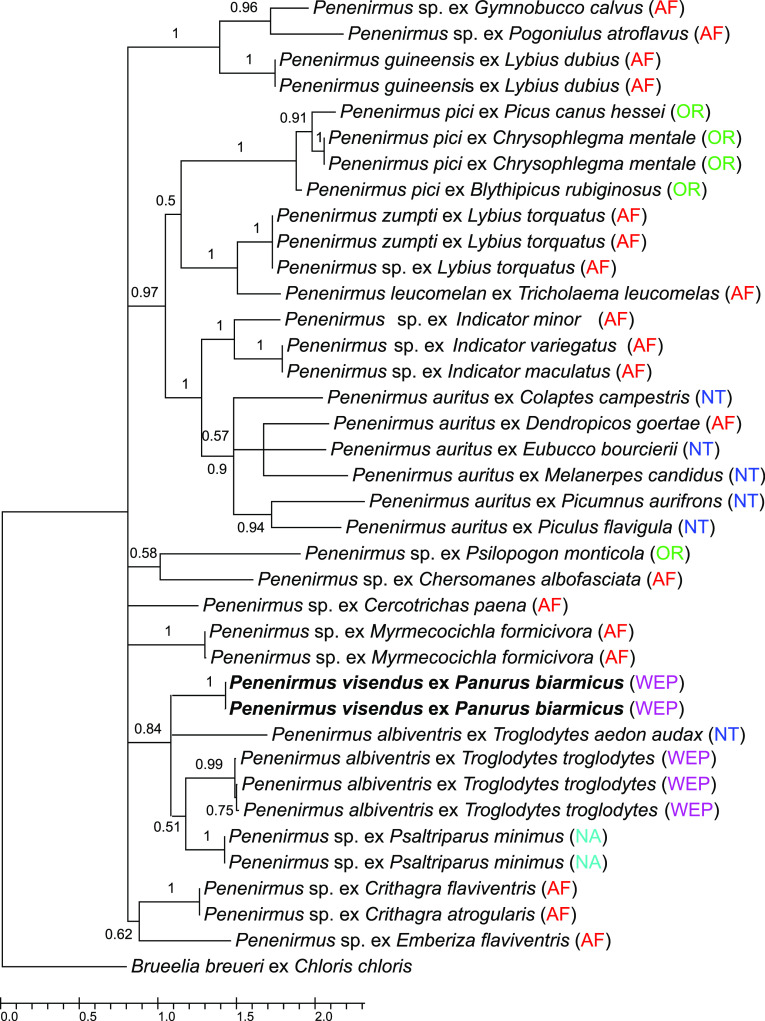
Phylogenetic tree of the *Penenirmus* species based on partial *COI* sequences, estimated with Bayesian analysis based on a 379 bp alignment of a *COI* gene fragment. The tree is drawn to scale, with branch lengths measured in the number of substitutions per site. The numbers above the branches indicate Bayesian posterior probabilities. Branches with posterior probabilities < 0.5 were collapsed. New sequences are in bold type. The origin of the samples is in parentheses: AF – Afrotropical realm; NA – Nearctic realm; NT – Neotropical realm; OR – Oriental realm; WPA – Western Palearctic realm.

A fragment of the *COI* gene was sequenced from four specimens of *P. visendus* (GenBank accession numbers OR533291–OR533292). Comparing our sequences with other available sequences from *Penenirmus* genus, the closest were those of *P. albiventris*, with sequence divergences around 18–19%. These sequence divergences are large enough to confirm *P. visendus* as a separate species, at least until sequences from other species are known. These data support the aforementioned morphological differences. Phylogenetic relationships among sequences obtained from *P. visendus* and sequences from other *Penenirmus* species are presented in Figure 7.

**Male** (*n* = 20). As in Figures 4A and 6D. Head (Fig. 5A) with postantennal suture, with one post-nodal and three post-temporal setae on each side, all of them short and spine-like. Marginal temporal setae 1 and 3 long, other marginal temporal setae short. Anterior dorsal setae of forehead shorter than distance between them. Dorsal anterior head plate quite large with slightly concave anterior margin and short blunt posterior process (Fig. 5B). Metanotum and metapleurite with an almost continuous row of 7 evenly spaced setae on each side (outmost lateral short metapleural seta included). Mesosternal plate with 2 setae, metasternal plate with 4 setae.

Tergites II–VI with anterior median notches, joined by two narrow conspicuously pigmented strips. Postspiracular setae on tergites III–VII long (0.28–0.33). Posterocentral tergal setae: II, 5–6; III, 6–7; IV, 6–7; V, 6–7; VI, 5–7; VII, 2–4; VIII, 2; IX, 4–6. Sternites lightly sclerotized with almost inconspicuous lateral plates. Sternal setae: II, 5–6; III, 9–12; IV, 10–12; V, 8–10; VI, 7–8; VII, 2. Paratergal setae: II–III, 0; IV–V, 1; VI–VII, 2; VIII–IX, 3. Genitalia as in Figure 5D.

Dimensions: HL = 0.38–0.49; POW = 0.29–0.37; HW = 0.35–0.48; PW = 0.22–0.31; PTW = 0.31–0.48; AW = 0.40–0.68; GW = 0.07–0.10; TL = 1.50–2.05.

**Female** (*n* = 20). As in Figures 4B,  6A and 6C. As for male, except as follows: head with only one short spine-like post-temporal setae on each side.

Tergites II–VIII with anterior median notches. Postspiracular setae 0.31–0.37 long. Posterocentral tergal setae: II, 6–8; III, 5–8; IV, 7–10; V, 6–9; VI, 6–8; VII, 6–7; VIII, 4; IX, 2. Sternal setae: II, 6; III, 7–10; IV, 8–11; V, 8–9; VI, 7–9; VII, 2; VIII, 2. Subvulval sclerites well-developed. Subgenital plate wide and slightly convex posteriorly, with 25–30 fine and 8–10 very short spine-like setae (Fig. 5C).

Dimensions (data for the holotype are in parentheses): HL = 0.42–0.51 (0.45); POW = 0.32–0.41 (0.33); HW = 0.41–0.52 (0.44); PW = 0.24–0.33 (0.24); PTW = 0.35–0.56 (0.42); AW = 0.54–0.92 (distorted); TL = 1.86–2.05 (1.95).

**Examined material. Holotype** ♀ ex *Panurus biarmicus*, Górki Wschodnie near Gdańsk, Poland, no. 9/c/1, leg. Zajac (MNHW No 786, Fig. 6B).

Other material. Non-types ex *P. biarmicus*: 5♂♂, 22♀♀ and 1 nymph, Velký Dvůr near Pohořelice (south Moravia), Czechia, 15 Jun. 1961, Balát’s collection no. FB1226, 1227, 1228, 1392 (MMBC); 2♂♂ and 2 nymphs from the same host, Neusiedl am See, Austria, 17 and 18 Sep. 1960 (FB1231, 1236), leg. Balát (MMBC); 20♂♂, 20♀♀, Gbelce, Slovakia, 20–30 Apr. 2009, 16–18 Jul. 2019, leg. Sychra & Ošlejšková (UVSB).

Note: According to Balát’s notes, there should be six items with *P. visendus* in his collection at the MMBC, under the numbers FB1226–28, 1231, 1236, and 1392. A total of 69 *P. visendus* had been collected from two males (single ♀ as item no. 1392 and 3♂♂, 12♀♀ and 14 nymphs as item no. 1228) and one female of *P. biarmicus* (6♂♂, 9♀♀ and 24 nymphs as item nos. 1226–27) examined on 15 June 1961 at Velký Dvůr near Pohořelice (south Moravia), Czechia. Additional 2♂♂ (FB1231) and two nymphs (FB1236) had been collected from male (examined on 18 Sep. 1960) and female of *P. biarmicus* (examined on 17 Sep. 1960) in Neusiedl am See, Austria, respectively. Balát considered these lice a new species, because he noted that item nos. 1226 and 1227 contain the holotype and paratype, respectively. However, he never formally described them. Despite Balát’s notes, not all aforementioned lice were mounted on slides, so at present there is a total of 7♂♂, 22♀♀ and 3 nymphs of *P. visendus* on 19 slides with numbers FB1226 (1 slide), 1227 (6 slides), 1228 (9 slides), 1231 (1 slide), 1236 (1 slide), and 1392 (1 slide) deposited at the MMBC. All other specimens are missing from the MMBC and must be regarded as lost.

## Discussion

The avifauna of the reed beds represents a mixture community of both habitat specialists that breed in this environment, as well as bird species that only roost in reed stands during both the breeding season and migration. In the case of habitat specialists, Sychra *et al.* [51] found significantly higher prevalences and mean abundances of chewing lice on resident and short-distance migrants (*Acrocephalus melanopogon*, *Panurus biarmicus*) than on long-distance migratory species (*Acrocephalus scirpaceus*, *A. schoenobaenus*, *Locustella luscinioides*). Except for the aforementioned birds, the highest infestations of lice in this study were found on birds roosting in reed beds in larger flocks, such as *Hirundo rustica* or *Sturnus vulgaris*. Horizontal transmission of lice is more frequent in communal roosting places where close contact between larger number of individuals can take place. Prevalence and intensity of lice are thus usually higher as well on these birds [27, 45]. We suggest that reed beds play an important role in the maintenance and dispersal of ectoparasites in the population of these hosts. On the other hand, despite large sample sizes, neither lice nor eggs were found on *Acrocephalus arundinaceus* (*n* = 107), even though *Menacanthus curuccae* and *Philopterus fedorenkoae* (Mey, 1983) are known from this host [35, 40].

In the present study, we evaluated 33 host-louse associations including 12 genera of lice. These associations include both 1) host-generalist, parasitizing more than one host species and host-specific lice, occurring only on single host species, and 2) lice species with broad geographic distribution, reported across the range of distribution of their hosts and lice species with only rare records in a limited area (for hosts see Price *et al.* [40]; for distribution see Mey [33], unless otherwise noted).

Among amblyceran lice, we found mainly host-generalist members of three genera that were also well recorded across Europe: *Menacanthus* – *Me. eurysternus* (reported from more than 170 hosts all around the world) [32], *Me. curuccae* and *Me. sinuatus* (Burmeister, 1838) (13 and 8 hosts, respectively in the Palearctic and Nearctic realms); *Myrsidea – My. cucullaris* (Nitzsch, 1818) (2 hosts in the Palearctic realm), *My. rustica* (3 hosts all around the world), and *My. quadrifasciata* (Piaget, 1880) (33 hosts all around the world) [50]; and *Ricinus – R. fringillae* De Geer, 1778 (47 hosts all around the world) [43].

An example of a widely distributed host-specific louse species is *Myrsidea latifrons* (Carriker [& Shull], 1910) that was reported from its host *Riparia riparia* (Linnaeus, 1758) across the world, including most places in Europe (Kolenčík & Sychra, unpublished data).

Among rare species, we can name one host-generalist: *Menacanthus chrysophaeus *(Kellogg, 1896), and three host-specific species: *Machaerilaemus clayae* (Balát, 1966), *Menacanthus brelihi Balát, 1981*, and *Menacanthus obrteli* Balát, 1981.

*Menacanthus chrysophaeus* was originally described from six hosts in the Nearctic realm [39], but recently it was also reported from *Emberiza schoeniclus* (Linnaeus, 1758) from Turkey, Greece, and Spain [5].

*Machaerilaemus clayae* is another parasite of *Riparia riparia*, but contrary to *Myrsidea latifrons* there are only a few records of this species from Czechia [3], Romania [1], Moldova, and Russia (Volga-Kama Nature Reserve) [12].

*Menacanthus obrteli* was originally described by Balát [4], but this name was later synonymized with *Me. takayamai* Uchida, 1926 [40]. Nevertheless, Sychra *et al.* [47] recently confirmed *Me. obrteli* as a valid species with *Locustella luscinioides* as the only host (see also Martinů *et al.* [32]). To date, there are only a few records of this louse from Czechia [4, 47], Slovakia [36], and Hungary [54].

We can see the same scenario for *Menacanthus brelihi* from *P. biarmicus*. This species was originally described by Balát [4]. Consequently, Krištofík [29] synonymized *Me. brelihi* with *Me. eurysternus.* After examination of type material and newly collected material we can confirm Balát′s assertion. Analysis of the *COI* gene shows that *Me. brelihi* is close to *Me. takayamai*, but the high divergence of sequences of these two species (14–16%) confirms *Me. brelihi* is a separate species. Moreover *Me. takayamai* seems to be paraphyletic and additional molecular analyses including more genes are needed to confirm relationships within this complex of species. To date, *Me. brelihi* has been reported from *P. biarmicus* from Czechia and Austria [4], and from Romania [42], and based on our sampling also from Slovakia [36, 51].

In the case of ischnoceran lice, we found several host-generalists that are also well recorded across Europe: *Acronirmus gracilis* (reported from 12 hosts all around the world) [14], *Penenirmus auritus* (Scopoli, 1763) (52 hosts all around the world), *Penenirmus albiventris* (2 hosts in the Palearctic, Nearctic, and Neotropical realms) [49], *Philopterus citrinellae* (Schrank, 1776) (16 hosts in the Palearctic realm) [37], *Rostrinirmus ruficeps* (Nitzsch [in Giebel], 1866) (5 hosts in the Palearctic, Oriental, and Afrotropical realms) [40].

Examples of widely distributed host-specific lice are *Brueelia nebulosa* (Burmeister, 1838) and *Sturnidoecus sturni* (Schrank, 1776) that are reported on the host *Sturnus vulgaris* from most areas of Europe, and *Rallicola cuspidatus* (Scopoli, 1763) as a specific parasite of *Rallus aquaticus*.

Among rare species, we can name two host-generalists: *Philopterus acrocephalus* Carriker, 1949 (5 hosts in the Palearctic realm) [35], and *Philopterus microsomaticus* Tandan, 1955 (3 hosts; reported in Europe only from Finland and Poland); and four host-specific species: *Brueelia blagovescenskyi* Balát, 1955 (in Europe reported from Czechia, Germany, Hungary, and Spain), *Brueelia locustellae* Fedorenko, 1975 (in Europe reported from Germany and Ukraine), *Brueelia vaneki* Balát, 1981 (reported only rarely from Czechia and Slovenia) [14, 15], and *Penenirmus visendus*. To date, *P. visendus* has been reported only from Poland [55] and Romania [42], and based on our sampling also from Slovakia [36, 51]. Undetermined species of *Penenirmus* have also been reported from Hungary by Rékási [41]. We extend the area of ​​distribution of this louse species to include Austria and Czechia.

In our study, *Panurus biarmicus* has fragmented distributions in Central Europe. Bush *et al.* [9] showed that habitat fragmentation may impact the prevalence of lice. The low infestation indices of *Menacanthus brelihi* may follow a similar scenario, i.e., smaller populations of hosts on the edge of their range may harbor fewer lice [38]. Sampling of *P. biarmicus* in other parts of its range is necessary to confirm that the rareness of this species is related to habitat fragmentation.

On the other hand, our results show that *Penenirmus visendus* is a common parasite of *P. biarmicus*, occurring with quite high prevalence throughout year [51]. Therefore, we suppose that *P. visendus* could have a stronger impact on its host than *Me. brelihi*. However, we cannot exclude that *P. visendus* is, on the contrary, a less virulent parasite that can occur in higher numbers without having a significant effect on the condition of its host. An experimental study is necessary to evaluate these assumptions. The present study also demonstrated the importance of accurate identification of parasites, especially on rarely examined and endangered species, where the knowledge of parasite diversity can be useful in their conservation programs. Moreover, Gustafsson *et al.* [16] suggested that parasites on endangered hosts, especially those that are host-specific, should also be treated as endangered. *Panurus biarmicus* is evaluated as Near Threatened in Slovakia [10], so if we adopt the idea of Gustafsson *et al.* [16], then both louse species on this host, but especially *Me. brelihi*, may be considered to have the same conservation status.

## Supplementary material

The Supplementary materials of this article are available at https://www.parasite-journal.org/10.1051/parasite/2024006/olm.
